# Supplementary material for: Strong influence of north Pacific Ocean variability on Indian summer heatwaves
Source: Nat Commun. 2022 Sep 12;13:5349. doi: 10.1038/s41467-022-32942-5 (PMC9468140; doi:10.1038/s41467-022-32942-5)
Supplement: Supplementary file 1 — Supplementary Information [file 41467_2022_32942_MOESM1_ESM.pdf]

Supplementary Material for

Strong influence of north Pacific Ocean variability on Indian summer heatwaves

Vittal Hari[1,2*], Subimal Ghosh[3,4], Wei Zhang[5], and Rohini Kumar[2,*]

[1]Indian Institute of Technology (Indian School of Mines), Dhanbad – 826004, India

[2]UFZ-Helmholtz Centre for Environmental Research, Leipzig, 04318, Germany

[3]Department of Civil Engineering, Indian Institute of Technology Bombay, Mumbai – 400076, India

[4]Interdisciplinary Program in Climate Studies, Indian Institute of Technology Bombay, Mumbai – 400076, India

[5]Department of Plants, Soils and Climate, Utah State University, Utah, USA.

[*]Corresponding Authors: Vittal Hari (vittal@iitism.ac.in; vittal.hari@ufz.de) and Rohini Kumar (rohini.kumar@ufz.de)

Supplementary Note 1: Mortality analysis

Mortality data from 1967–2010 is obtained from the India Meteorological Department and from annual reports, which compiled information from newspaper and other sources about mortality during specific extreme heat events[1]. The detailed information pertaining to this mortality data is available in Mazdiyasni et al[1]. The major heatwave years, along with their time of occurrence are further procured from Mishra et al[2]. They estimate these aspects using the method proposed by Russo et al[3]. The heatwave magnitude index daily (HWMId), as described by Russo et al[3], provides a basis to understand severity (e.g. magnitude) and duration of heatwaves in any given region. The HWMId is based on the maximum magnitude of heatwaves in each year, where a heatwave is defined as a period with T_{max} above a daily threshold for three or more consecutive days. Readers are informed to refer Mishra et al[2] for more details.

Supplementary Note 2: Estimation of risk to heatwaves

Here, we estimate risk based on the definition following the IPCC-AR5 framework[4] – i.e. **Risk** $=$ **Hazard** $\times$ **vulnerability**[5], with exposure component included in the vulnerability section itself[6]. In case of heatwave **hazard**, we estimate the trend in TXx intensities from 1951–2019 based on a gridded daily maximum temperature data from India meteorological department[7], which is available at a 1° spatial resolution.

The **vulnerability**, on the other hand, is estimated based on a demographic data procured from Census of India for the decade 2011, which basically provides decadal information on different categories of population. A total of 35 relevant indicators are selected to reflect an impact on the socio-economic system across India (see Vittal et al[6] for complete description on these indicators). These indicators are standardized mainly

to make the indicators dimensionless[8,9], which allow us to compare the different indicators at a national scale[10]. We then perform a Principal Component Analysis (PCA) to decorrelate and reduce the dimensionality; and select the Principal Components (PCs) explaining 75% of the variability in indicators. These PCs were then considered as input in a robust non-parametric data envelopment analysis (DEA)[11] framework to obtain the vulnerability rankings of each decision-making unit (DMU), which, in this case, were the districts of India[6,12]. The detailed information pertaining to the vulnerability estimation can be obtained from Vittal et al.[6].

The gridwise trend values of heatwave hazard component are aggregated at a district-scale. Finally, the risk map is derived based on these information in the form of bivariate mapping (shown in Fig. 1b) – as this can provide an effective understanding of the individual and combined/joint intensity of both the components (vulnerability and hazard).

Supplementary Note 3: Climate model perturbation experiment

To evaluate the impacts of the SST anomaly on NCI heatwaves and large-scale circulation, we performed two sets of perturbation experiments which are integrated for 30 years. Here we use atmospheric general circulation model developed by International Centre for Theoretical Physics (ICTP AGCM)[13,14], which could properly capture the observed pattern of climatology over India[15]. Initially, we start the experiment by prescribing the seasonal climatology of SST based on ECMWF reanalysis (CLIM; averaged from 1979–2008). Further, to evaluate the response of NCI heatwaves, the observed composite anomaly of SST during positive phase of PMM over the PMM region (Supplementary Fig. 9a) and El Niño over ENSO region (Supplementary Fig. 9c) for June are superimposed on the monthly

climatology, keeping SST identical to the CLIM experiment for other months. The subtraction of CLIM experiment with the SST anomaly forced experiment then provides the response of large-scale atmospheric circulation, to both PMM and El Niño.

Supplementary Note 4: Role of internal variability Along with these CMIP6 models, we also use the large-ensemble available from the MPI grand ensemble (MPI-GE; 100 ensembles) and MIROC6 CMIP6 climate model (50 ensembles) to assess the role of internal variability on the PMM and NCI relationship. The basis of the selection of this particular model is mainly based on the fact that MPI-GE offer the best global and regional representation of both the internal variability and forced response in observed historical temperatures compared to other CMIP5 large ensembles[16]. On the other hand, we select MIROC6 CMIP6 large ensemble because it generally falls under the observational constrained group of models. The MIROC6 has a climate sensitivity values that are within the IPCC AR5 likely range and also shows that warming trend is much more consistent with the observations, compared to other available large ensemble such as CanESM5[17]. Together with the large-ensemble of historical simulation, we also utilize the 500 years of control simulation for both MPI-GE and MIROC6.

Further we analyse on the PMM constraints in the future projections of temperature change over NCI, using MPI-GE. The MPI-GE showed that all the ensemble did represent the relationship between summer PMM and NCI temperature equally good – with very less internal variability in the future projections as well. From these ensembles, we select the members with the higher positive association with the PMM index – which is similar or above the observed value (based on method of Huang et al.[18]), and analyse the effect of PMM on the future projections. We notice a significant increase in the surface temperature (approximately 0.10 degree C) in these ensembles compared to the rest of the ensembles

(Supplementary Fig S14a and b). Similar analysis is performed from the 50 MIROC6 ensembles. Here, we select 10 members with the highest trends of temperature with PMM – which is near to the observed trend. Here also we notice a significant increase in the surface temperature (approximately 0.15 – 0.17 degree C) in these 10 ensembles compared to other ensembles (Supplementary Fig S14c and d), further supporting the results obtained from the group of selected individual CMIP6 models.

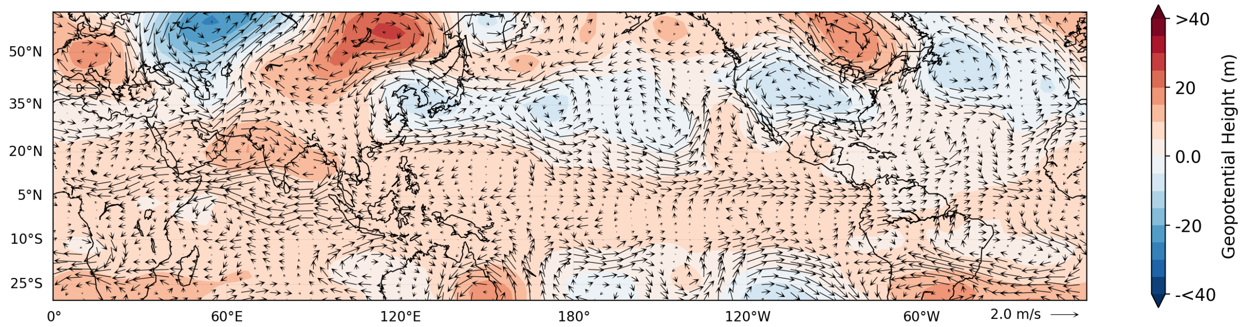

Figure S1: The Composite anomaly of geoptential height and winds at 500 hpa estimated during the heatwave summers.

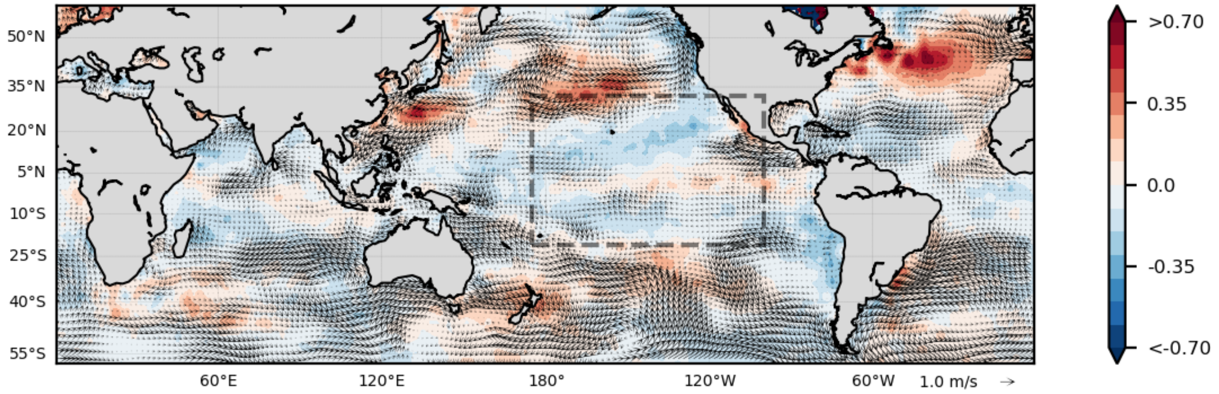

Figure S2: The Composite anomaly of SST (°C) and surface wind estimated during the non heatwave summers, wherein the black rectangular over tropical Pacific ocean regions between 175°E–95°W and 21°S–32°N represents the Pacific Meridional Mode (PMM) spatial structure during its negative phase.

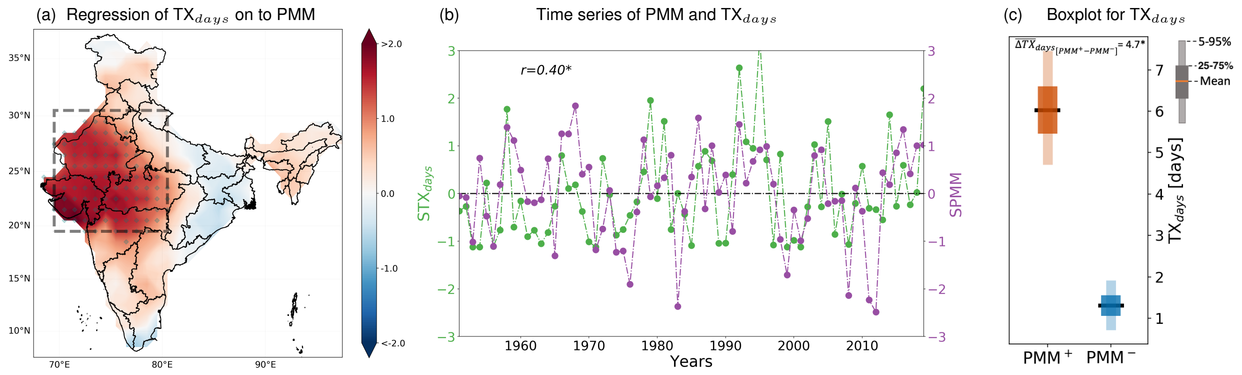

Figure S3: Regression of June heatwave indicator (in days; estimated with the 90 percentile threshold, estimated by considering the base period of 1961–1990), over India onto June PMM index during the period 1951–2019. The hatched areas represent the locations where the slopes are significant at the 5% level. (b) Inter-annual variability of the June heatwave indicator (in days) estimated over the north western Indian region (depicted by a black rectangular region in the panel a) and PMM index. We notice a prominent positive correlation between the PMM and heatwave over the North western Indian region, which is significant at 5% level (represented with "$*$" symbol). The years with standard deviation $>$ ($<$) 1 (-1) from this Inter-annual variability of PMM index is further considered as the positive (negative) phase of PMM and the heatwave indicator (days) during these phases are represented with the box plot (c). The differences are statisticall significant (based on non-parametric bootstrap analysis; p-value ≤ 0.05)

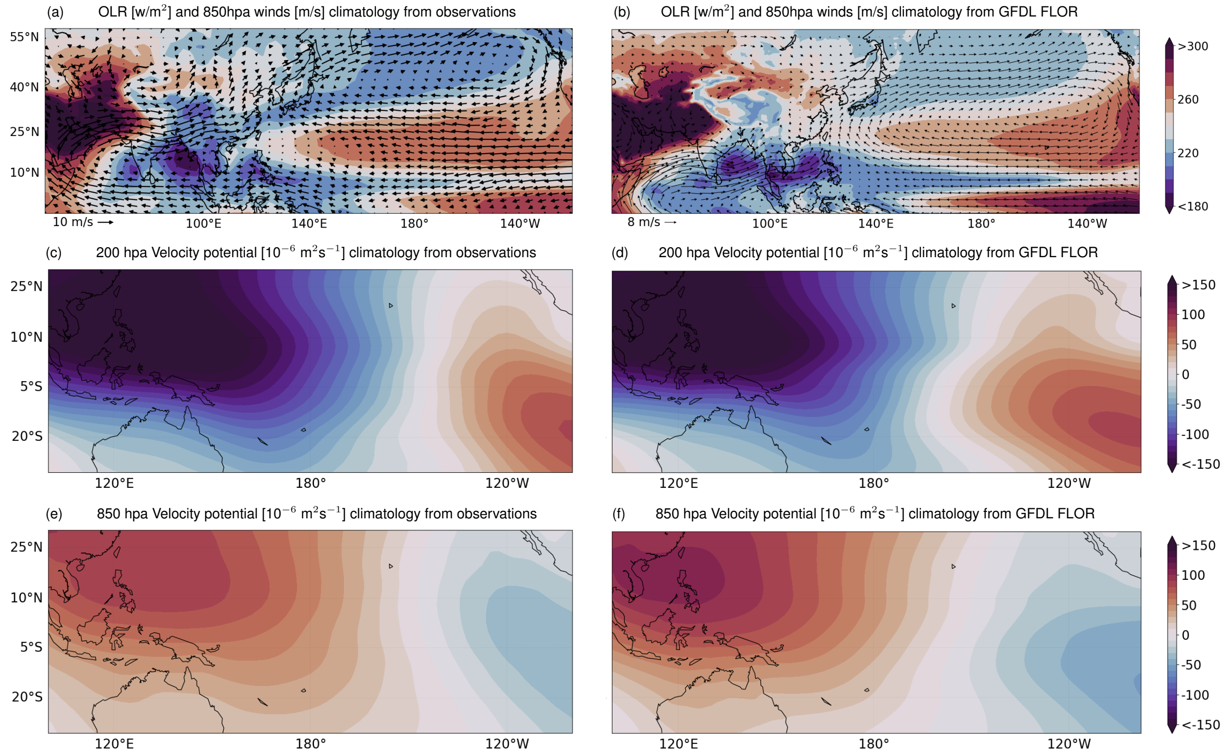

Figure S4: (a) and (b) Climatology of outgoing longwave radiation (OLR; in shading) and winds at 850hpa pressure level (vectors) from the NCEP and GFDL FLOR experiment datasets. (c) and (e) are the June climatology of velocity potential at 200 and 850 hpa levels from the reanalysis data. (d) and (f) is same as (c) and (e), but from the GFDL FLOR control experiment.

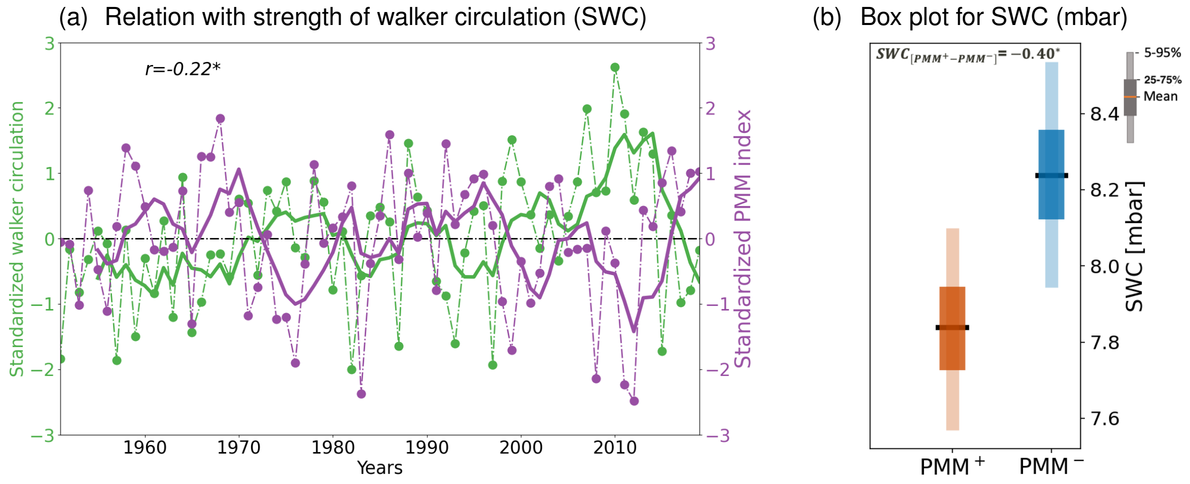

Figure S5: Relationship between strength of walker circulation with the PMM during June.(a) Inter-annual variability of the leading PMM index and strength of walker circulation (SWC), with variability being represented in terms of their standardized scores. Here, the SWC is estimated by sea level pressure anomaly differences across the Pacific, which is associated with vertical motions of the walker circulation[19]. The described region over the tropical Pacific is 4.74°S – 4.74°N in latitude, and 128.39°E – 151.05°E and 211.47°E – 231.61°E in longitude for the western and eastern tropical Pacific edge, respectively. We notice a significant negative correlation between the PMM index and SWC, (p-value ≤ 0.10; represented with "$*$" symbol). The years with standard deviation $>$ ($<$) 1 (-1) from this Inter-annual variability of leading PMM index is further considered as the positive (negative) phase of PMM and the SWC during these phases are represented with the box plot (b): the differences in SWC are statistically significant (non-parametric bootstrap analysis; p-value ≤ 0.10).

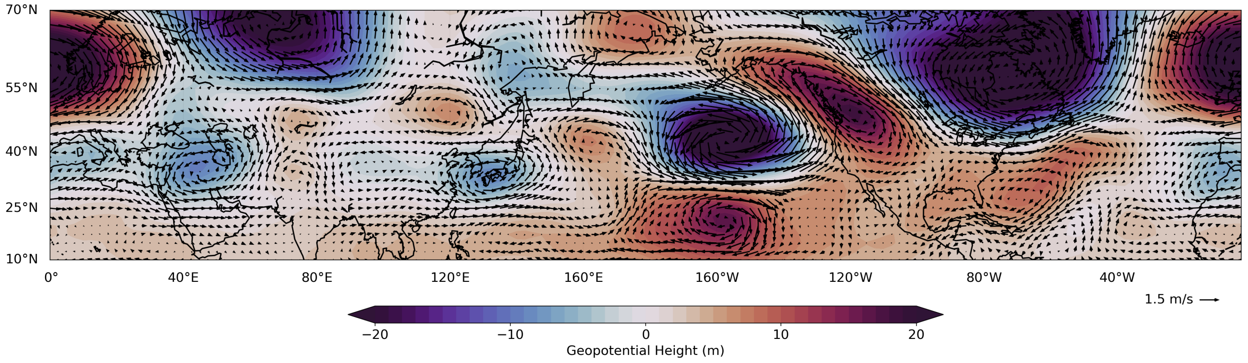

Figure S6: Large-scale atmospheric circulation pattern associated PMM in terms of the of geopotential height (shading) and wind (vectors) at 250-hPa level.

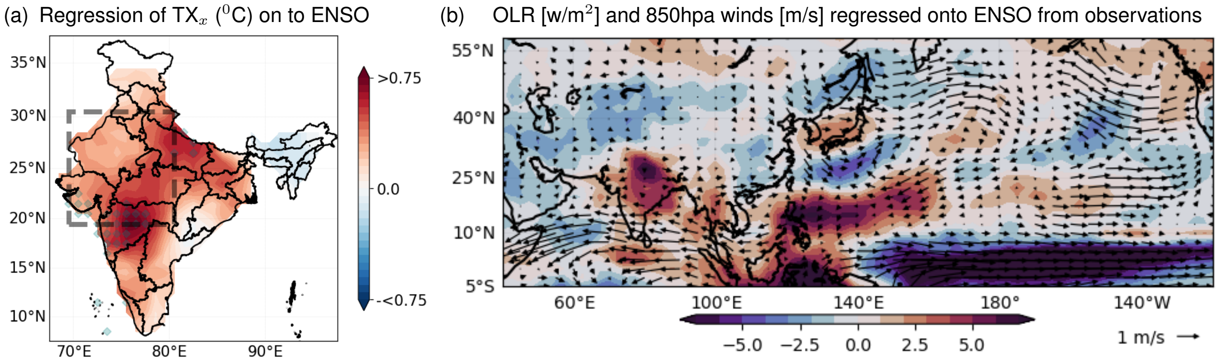

Figure S7: (a) Regression of TXx over India onto ENSO index (spatially averaged SST over the region 5°S–5°N and 170°W–120°W) during the June. The hatched areas represent the locations where the slopes are significant at the 5% level. (b) Large-scale atmospheric circulation pattern associated with the PMM in terms of the regression of outgoing long-wave radiation (OLR; shading) and winds at 850 hpa (vectors) from NCEP reanalysis. The analyses are performed for the period 1980–2019.

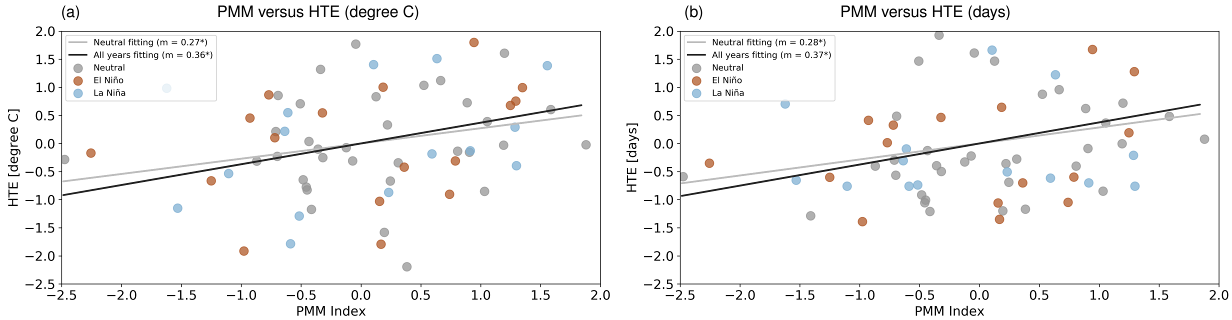

Figure S8: Scatter plots of PMM versus two heatwave indicators, viz., June three-day maximum (a) and June heatwave days (b) in NCI region during El Niño (red dots), La Niña (blue dots) and neutral ENSO years (black dots). Here, we exclude the June months that follow a strong El Niño and La Niña event in the previous three months (April-June), as suggested by Chiang and Vimont[20]. An El Niño/La Niña event is defined as the Niño3.4 index when it exceed 0.5℃ for atleast three consecutive months[20] (`https://origin.cpc.ncep.noaa.gov/products/analysis_monitoring/ensostuff/ONI_v5.php`). The fitted lines are computed for neutral ENSO years (grey line) and all years from 1951–2019 (black line). ”m” denotes the slope of these fitted lines and “*” symbol represent that these slopes are significant at 5% level.

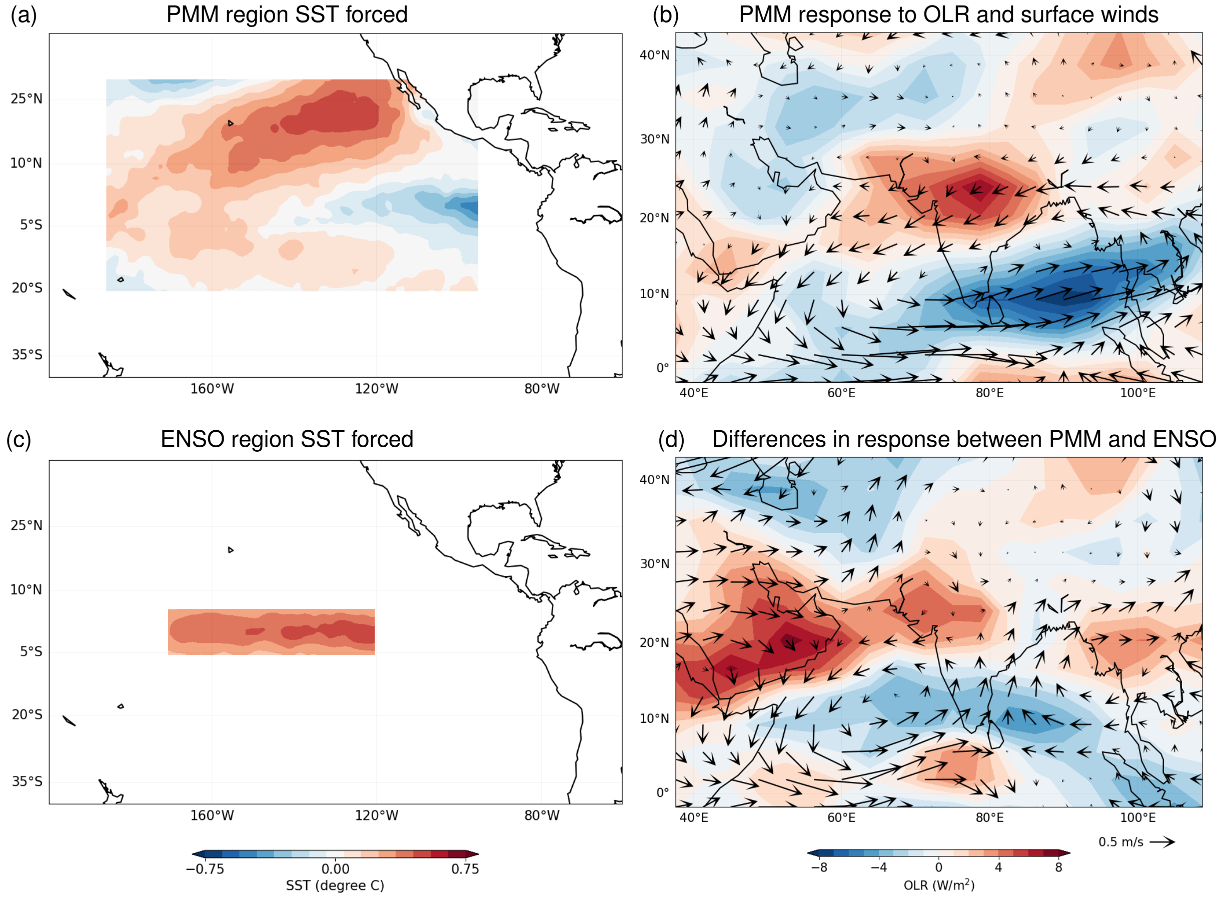

Figure S9: Sea surface temperature (SST) anomalies used in ICTP AGCM perturbation experiment for both PMM (a) and El Niño (c) phase. (b) Response of outgoing longwave radiation (OLR; in shading) and 850 hpa wind (vectors) to the perturbation experiment forced with PMM sst anomalies. These changes are estimated based on the seasonal climatology runs (CLIM). The differences in the response between the PMM and El Niño is provided in (d).

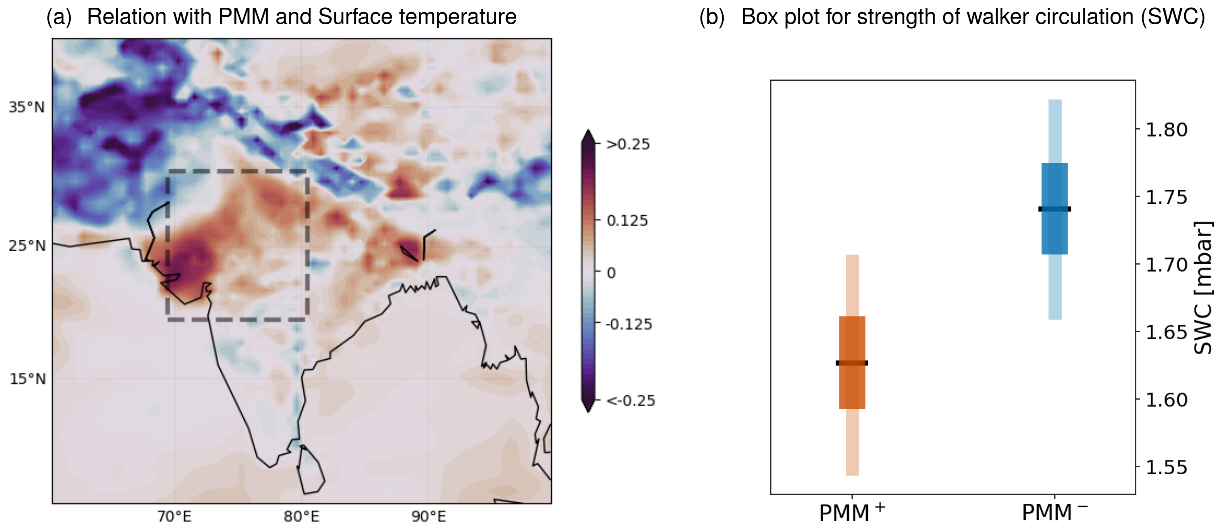

Figure S10: (a) Regression of June PMM index with the surface temperature considering GFDL–FLOR control runs. The black box in the plot represent the NCI region. (b) Response of strength of walker circulation (SWC) with respect to PMM; as shown in the difference between the positive and negative phase of PMM, which are represented with the box plot. The differences in SWC are statistically significant (non-parametric bootstrap analysis; p-value ≤ 0.05).

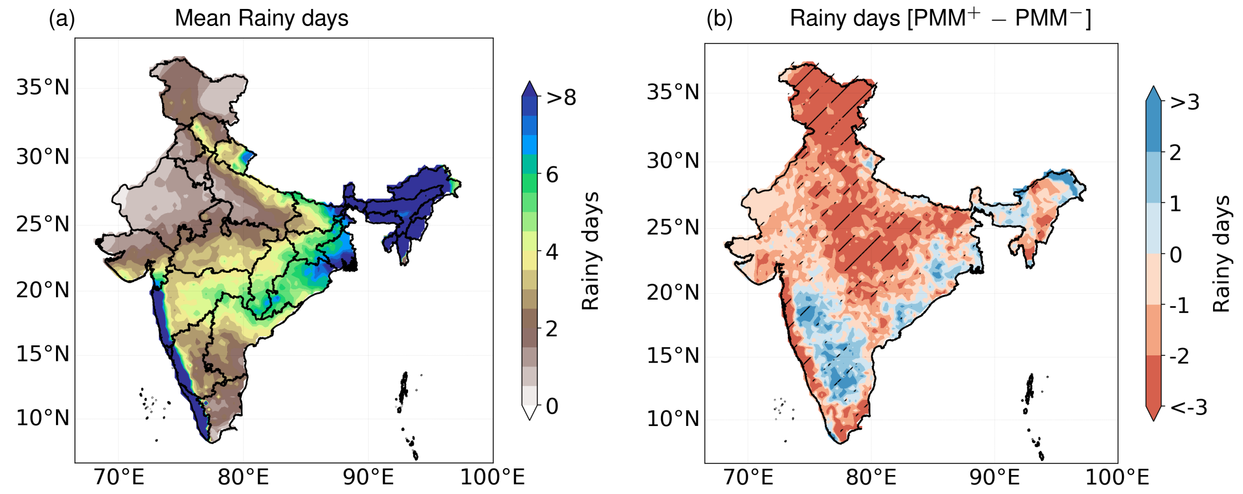

Figure S11: (a) Mean rainy days across India during early to mid June (covering first 3 weeks of the month). (b) Differences in rainy days between the years with strong positive and negative PMM phases. The hatched area represent that these changes are significant at 10% level.

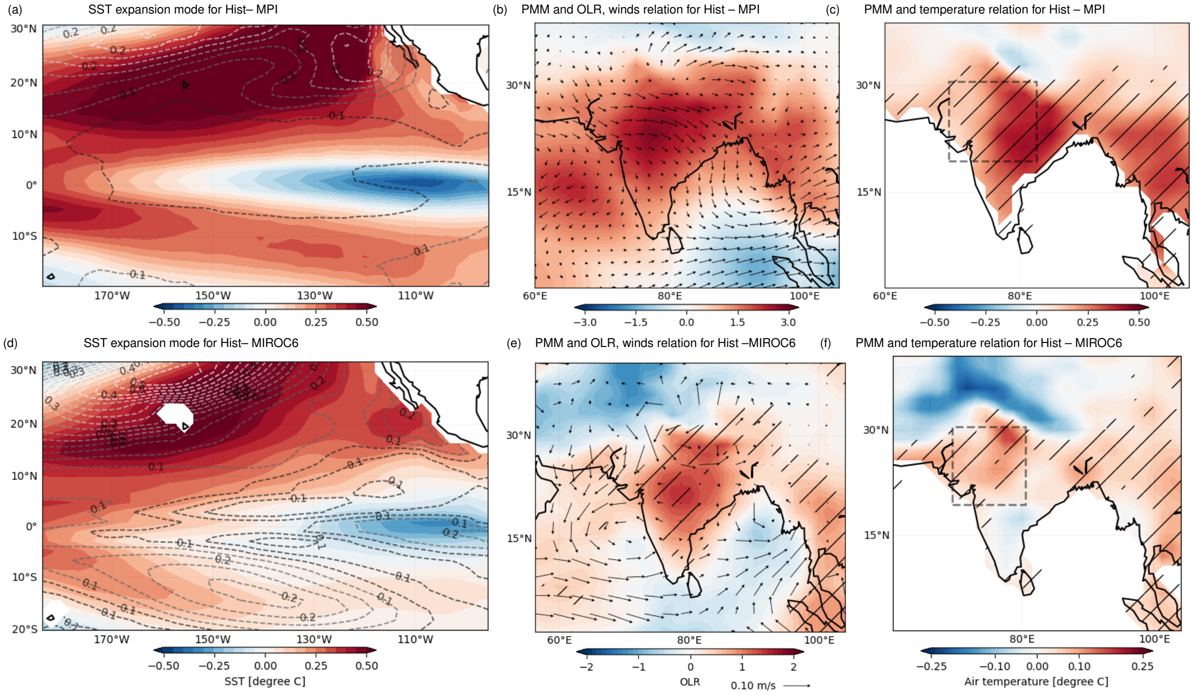

Figure S12: **Representation of PMM in the historical runs of large ensembles from MPI grand ensemble (MPI-GE) and MIROC6 CMIP6 climate model** (a) Shows the ensemble mean 100 members of the MPI-GE historical simulations for the leading coupled mode of SST and surface winds over the core PMM region during historical time period, i.e., 1951 – 2014. The contours represents the standard deviation of the SST leading mode amongst the ensembles members. (b) and (c) depicts the ensemble mean of the response of OLR, surface winds and surface temperature with the summer PMM index, respectively for MPI-GE. The stippling denotes at-least two-third (66%) of the 100 ensemble members agreement in signs. (d,e and f) is similar to (a, b and c) but for 50 member of MIROC6 large ensemble.

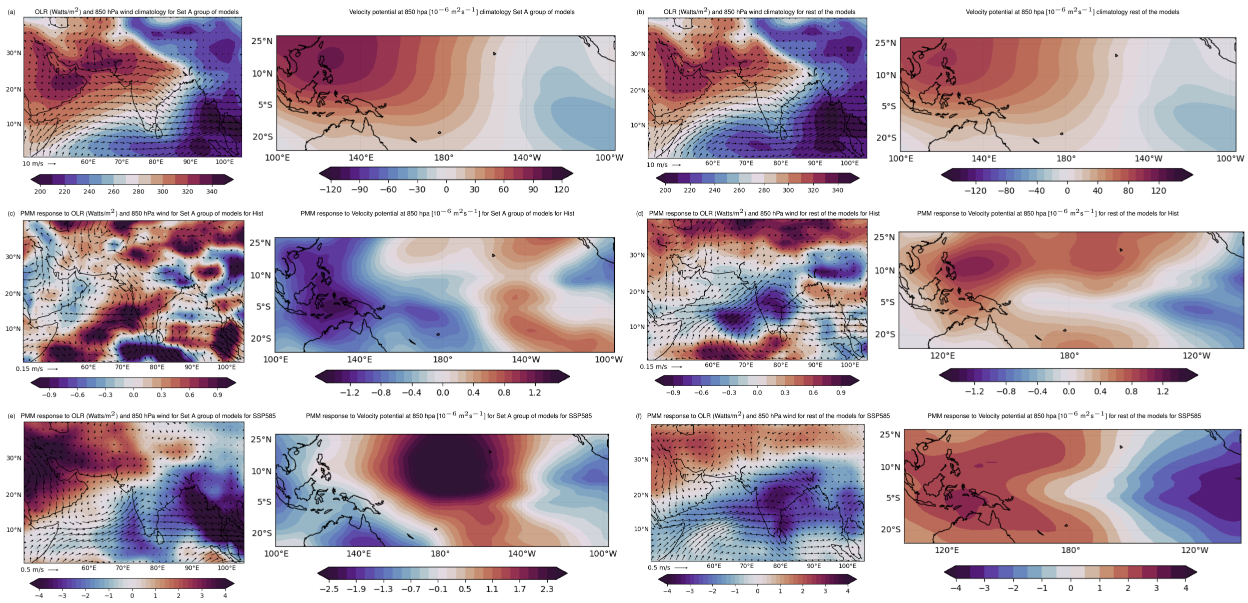

Figure S13: Ensemble mean of climatology of OLR and winds at 850 hPa level (a; left panel) and velocity potential at 850 hPa level (a; right panel) for the Set A group of models/realizations (which adequatly captures the PMM and NCI heatwave relationship) during the historical period (1951–2014). (c) and (e) shows the ensemble responses – in terms of regression analysis – of OLR and winds at 850 hPa level (left panel) and velocity potential at 850 hPa level (right panel) to PMM index during the historical and future period, respectively, for Set A group of models/realizations. The future period considered here is from 2065–2100 and for the SSP5-8.5 scenario. (b), (d) and (f) is same as (a), (c) and (e) but for rest of the CMIP6 models. Here, for the regression analysis for both during the historical and future period, we detrend the OLR, winds and velocity potential before we perform a regression analysis with PMM.

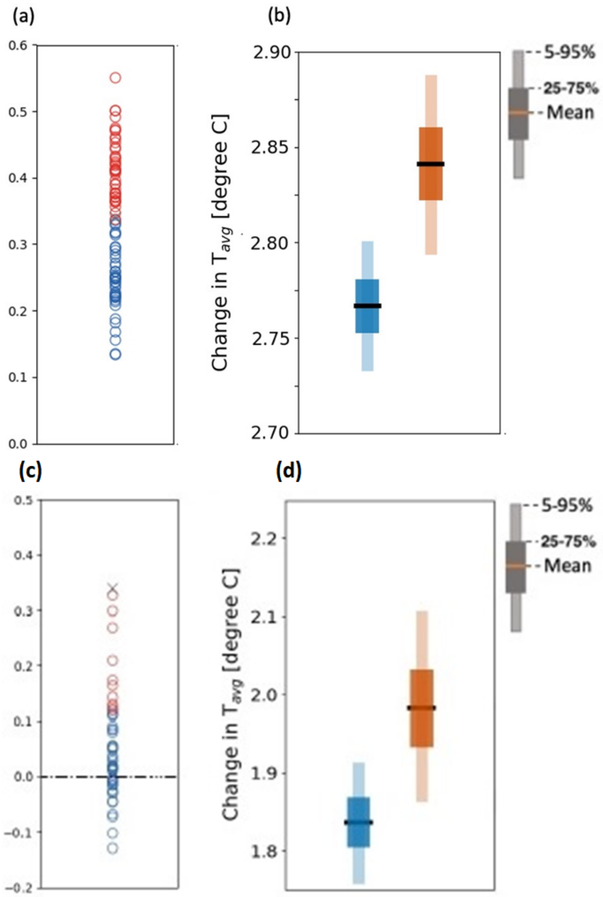

Figure S14: Evaluation of the historical PMM and NCI temperature in large ensemble. (a) The NCI temperature relation with the summer PMM index for the observed (black cross mark) and the MPI members (blue circles) during 1951-2014. The members with the higher positive association with the PMM index – which is similar or above the observed value – are shown in red circles. (b) Box-plots depicting the projected changes in the surface temperature intensities under RCP8.5 scenario over NCI region during the second half of the twenty-first century (2065–2100) w.r.t the historical simulations (1980–2014) for the members with the higher positive association with the PMM (red colored) and for the rest of the members (blue colored). (c) The NCI temperature relation with the summer PMM index for the observed (black circle) and the 50 MIROC6 members (blue circles) during 1951-2014. The 10 members with the higher positive association with the PMM index are shown in red circles. (d) Box-plots depicting the projected changes in the surface temperature intensities under SSP 5-8.5 scenario over NCI region during the second half of the twenty-first century (2065–2100) w.r.t the historical simulations (1980–2014) for the 10 members with the higher positive association with the PMM (red colored) and for the rest of the members (blue colored).

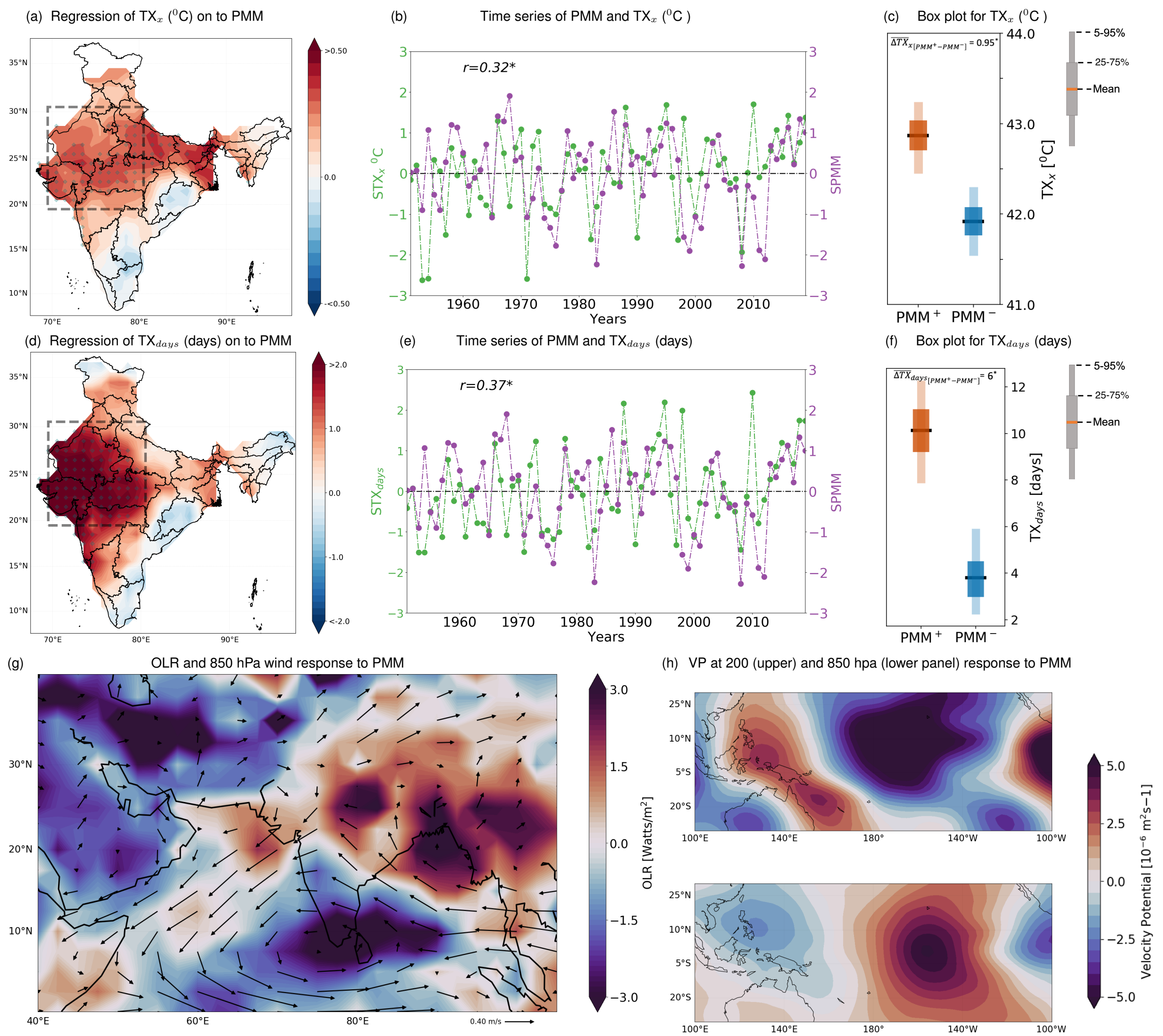

Figure S15: (a) Regression of May–June TXx over India onto leading PMM index during the period 1951–2019. The hatched areas represent the locations where the slopes are significant at the 5% level. (b) Inter-annual variability of the leading PMM index and May–June TXx estimated over the NCI region (black rectangular box in the panel a), with variability being represented in terms of their standardized scores. We notice a significant positive correlation between the PMM index and May–June TXx variability across NCI, (p-value ≤ 0.05; represented with "$*$" symbol). The years with standard deviation $>$ ($<$) 1 (-1) from this Inter-annual variability of leading PMM index is further considered as the positive (negative) phase of PMM and the TXx intensities during these phases are represented with the box plot (c): the differences in heatwave intensities are statistically significant (non-parametric bootstrap analysis; p-value ≤ 0.05).Bottom row (d,e and f) is similar to that of top row, but for the heatwave indicator (in days; estimated with the 90 percentile threshold, estimated by considering the base period of 1961–1990). (g) Large-scale atmospheric circulation pattern associated with the PMM during May in terms of the regression of outgoing long-wave radiation (OLR; shading during 1975–2019) and winds at 850 hpa (vectors, during 1951–2019) from NCEP reanalysis during May–June. The response of velocity potential at 200 (h; upper panel) and 850 (h; lower panel) hPa levels to the leading PMM index, wherein the negative (positive) values indicates the region with increased divergence (convergence).

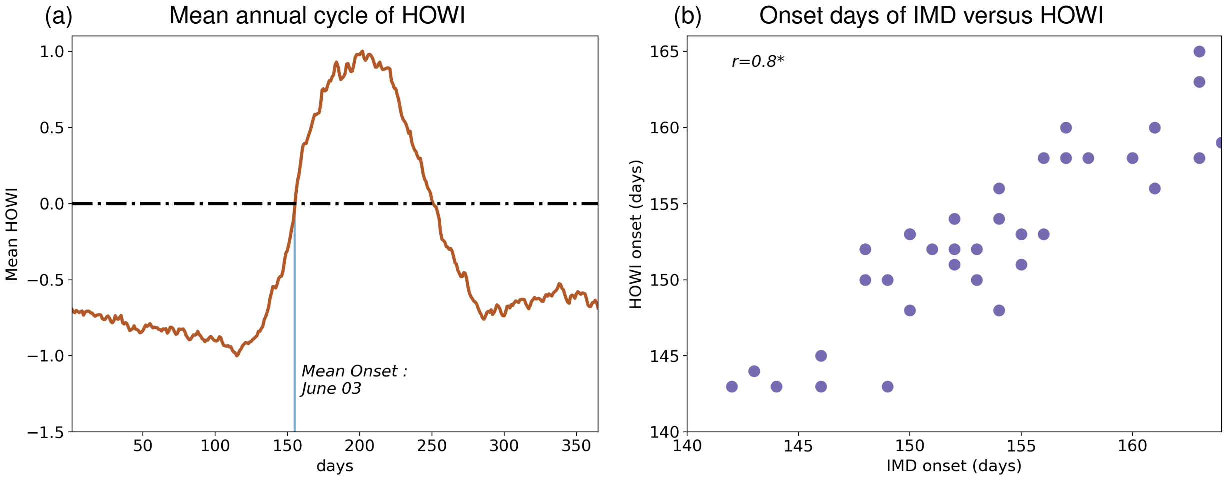

Figure S16: (a) Mean annual cycle of Hydrologic Onset and Withdrawal Index (HOWI).The HOWI onset dates are significantly correlated with the IMD onset dates (b).

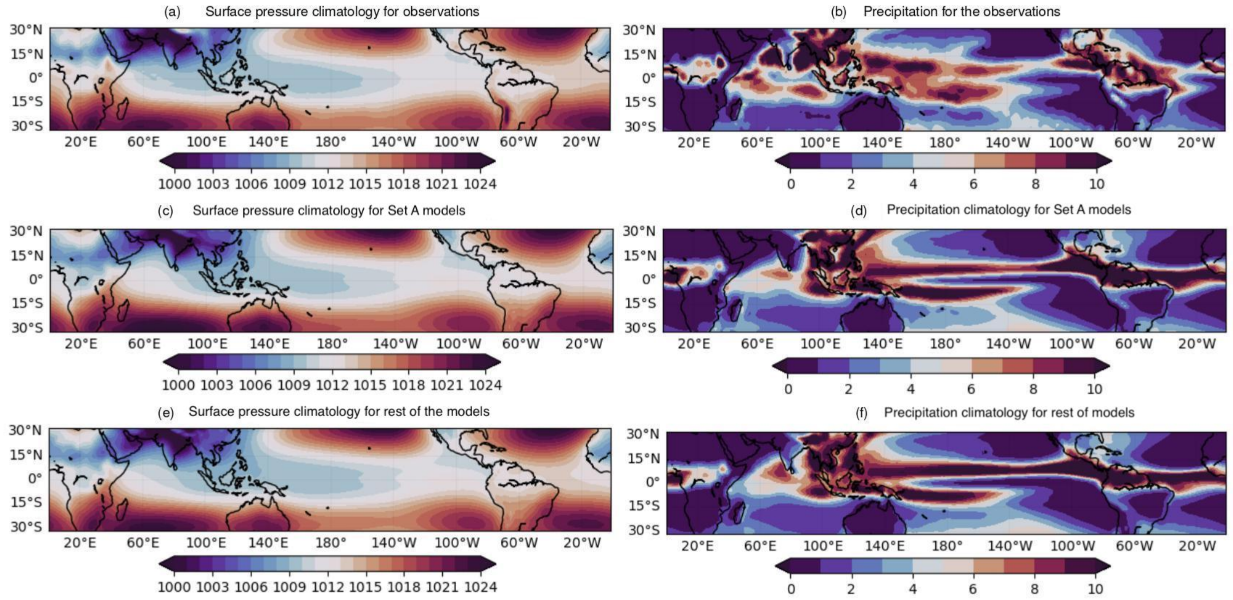

Figure S17: Basic states of the CMIP6 models in terms of surface pressure and topical rainfall during the month of June. (a) and (b) is the climatology of surface pressure and precipitation for the NCEP NCAR reanalysis data. (c) and (d) is the climatology for the set of Set A group of models/realizations. Shown are the ensemble mean of the climatology from the set of models. (e) and (f) are for the set of rest of models.

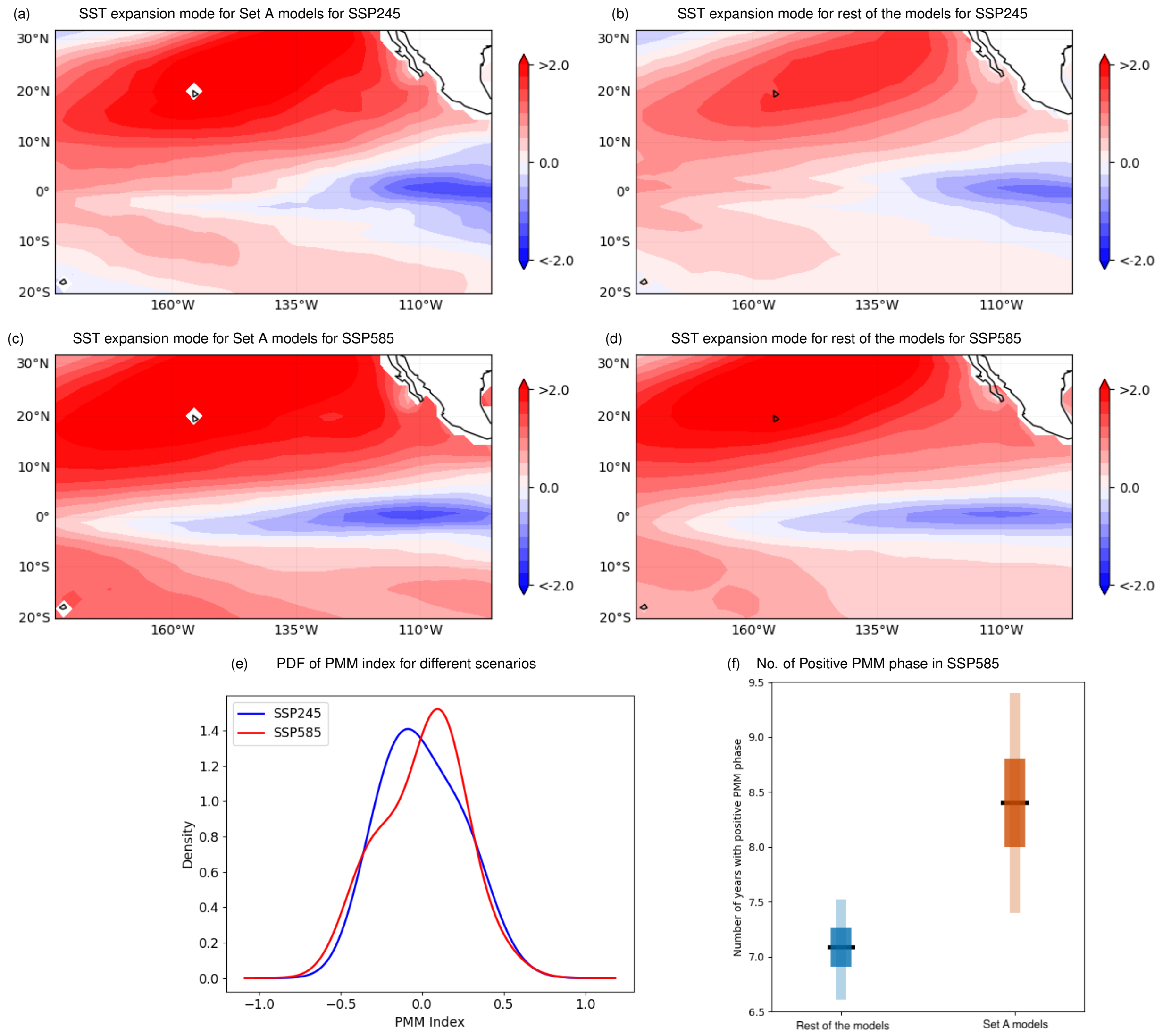

Figure S18: Representation of PMM in the state-of-the-art CMIP6 climate model simulations for different future scenarios. The leading coupled mode of SST and surface winds over the core PMM region, obtained from the maximum co-variance analysis. For depiction, we show the spatial patterns of SST expansion coefficient for (a) ensemble mean of Set A group of models/realizations and (b) ensemble mean of rest of the models for the medium emission scenario, SSP2-4.5. (c) and (d) is same as (a) and (b), but for the high emission scenario, SSP5-8.5. (e) The comparison of the PMM index for the future period, i.e., 2065–2100 for two emission scenarios (SSP2-4.5 and 5-8.5) are shown in the form of probability distribution function (PDF). (f) Depicts the box plot representing the difference in number of positive phases between Set A and rest of the models. The differences are significant at 5% level.

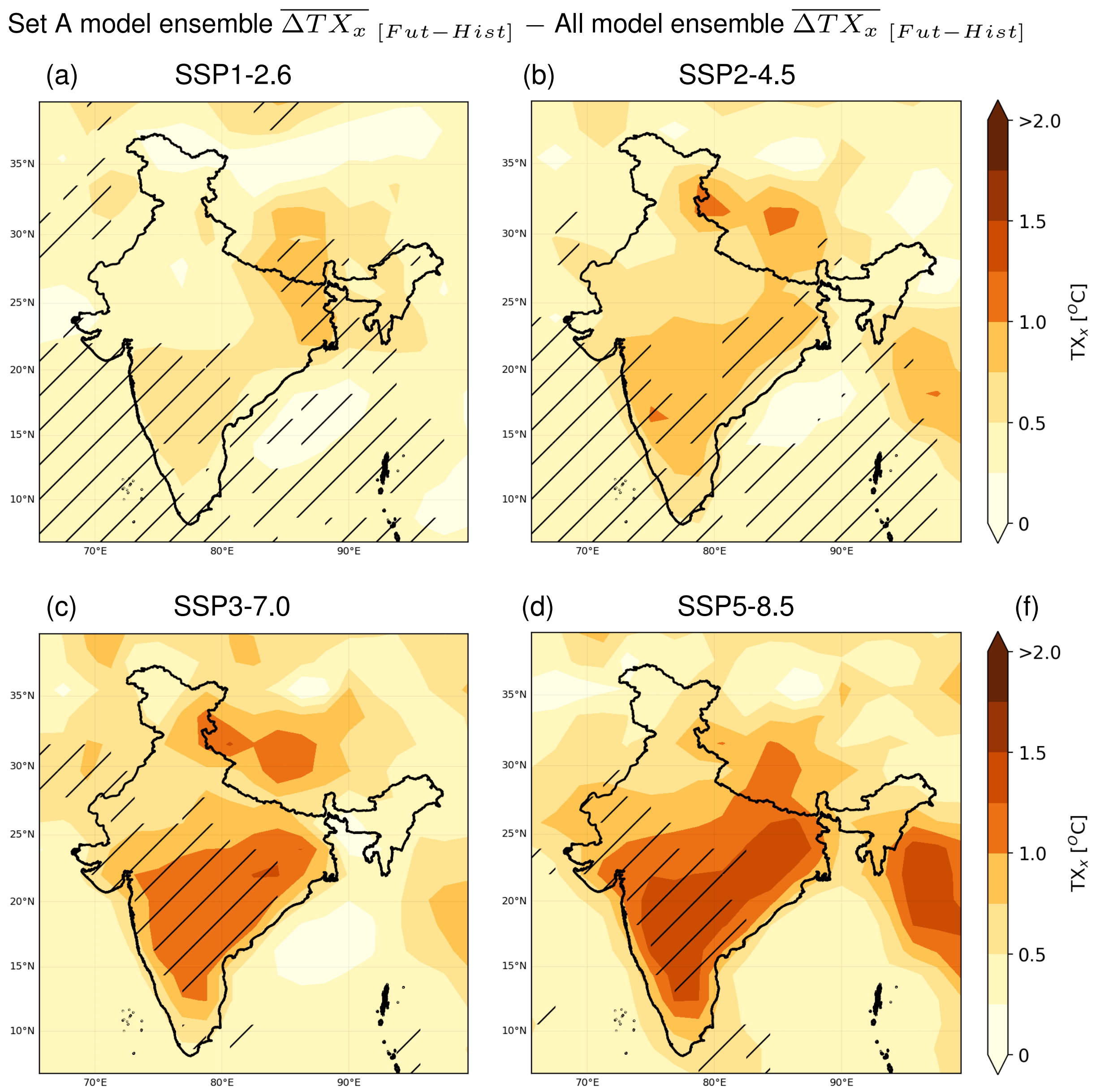

Figure S19: The difference in projected TXx [°C] intensities during the second half of the twenty-first century (2065–2100) w.r.t. the historical simulations (1980–2014) between Set A group of models/realizations and all model ensembles. The Set A group of models/realizations project more intense heatwaves compared to all model ensembles under different shared socioeconomic pathways (SSP) scenarios (a-d; See Methods). Hatched areas represent the locations where the difference of TXx intensities between the two model groups are significant at the 10% level.

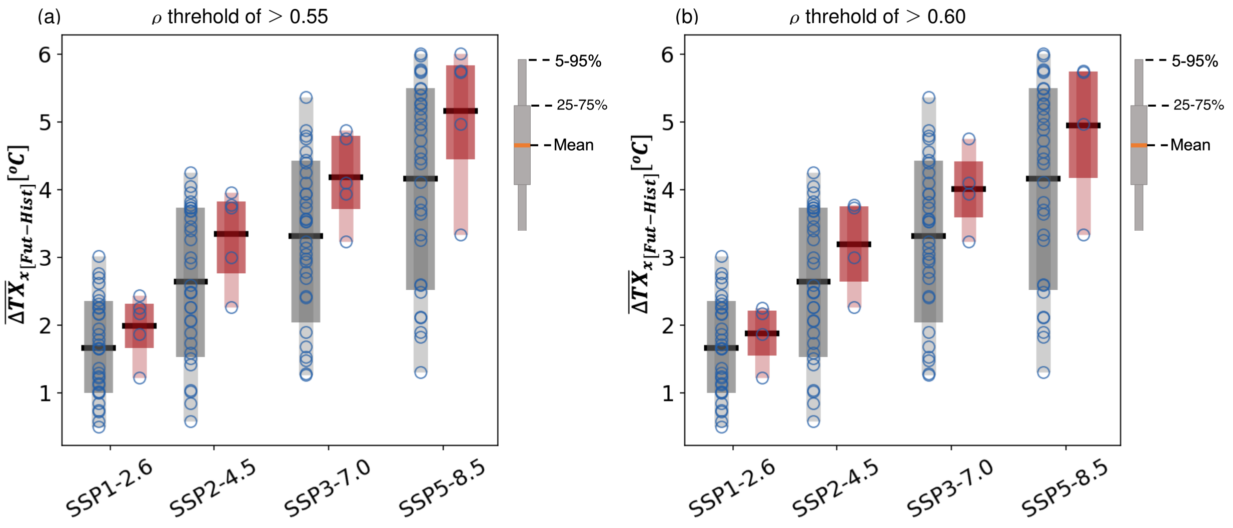

Figure S20: Relative changes in the TXx [°C] during the second half of the twenty-first century (2065–2100) compared to that of historical simulations (1980–2014) across different future projection scenarios considering all (gray) and Set A group of models/realizations (red) simulations. Here the Set A group of models/realizations are based on the rho threshold of $\geq$ 0.55 (a) and $\geq$ 0.60 (b). The blue open circles show individual model realizations considered for each groups.

Table S1: List of CMIP6 climate model ensembles used in the present study along with correlation values estimated between the simulated and observed spatial patterns of the leading PMM feature over the core PMM region and capturing the PMM response to the NCI heatwave variability in the historical period. See Methods and the related texts (Fig. 4) in the main manuscript for more details. All CMIP6 models were brought to a common spatial resolution. '*' indicates the Set A group of model/realizations. The observed $\overline{\Delta TX}_{XPMM^{+}-PMM^{-}}$ with the similar thresholds for positive and negative PMM phase is 1.4 °C

Sl. No.	Model	Variant	Correlation	$\overline{\Delta TX}_{XPMM^{+}-PMM^{-}}$ [°C]
1	ACCESS-CM2	r1i1p1f1	0.52	0.03
2	ACCESS-CM2*	r2i1p1f1	0.62	0.52
3	ACCESS-CM2*	r3i1p1f1	0.56	0.54
4	ACCESS-ESM1-5	r1i1p1f1	0.52	0.10
5	ACCESS-ESM1-5	r2i1p1f1	0.54	0.17
6	ACCESS-ESM1-5*	r3i1p1f1	0.53	1.21
7	BCC-CSM2-MR	r1i1p1f1	0.03	-0.80
8	CanESM5	r1i1p1f1	0.64	0.02
9	CanESM5*	r2i1p1f1	0.63	0.93
10	CanESM5	r3i1p1f1	0.69	0.24
11	CNRM-CM6-1	r1i1p1f2	0.25	-0.37
12	CNRM-ESM2-1	r1i1p1f2	0.18	-0.22
13	EC-Earth3	r1i1p1f1	0.49	-0.63
14	EC-Earth3*	r4i1p1f1	0.68	0.51
15	EC-Earth3-Veg	r1i1p1f1	0.53	-0.95
16	EC-Earth3-Veg	r2i1p1f1	0.64	0.06
17	EC-Earth3-Veg	r3i1p1f1	0.23	0.26
18	INM-CM4-8	r1i1p1f1	0.59	-0.41
19	INM-CM5-0	r1i1p1f1	0.46	0.44
20	IPSL-CM6A-LR	r2i1p1f1	0.22	0.42
21	IPSL-CM6A-LR	r3i1p1f1	0.72	-0.04
22	KACE-1-0-G	r1i1p1f1	0.61	-1.53
23	KACE-1-0-G	r2i1p1f1	0.58	-0.20
24	KACE-1-0-G	r1i1p1f1	0.50	-1.12
25	MPI-ESM1-2-HR	r1i1p1f1	0.61	0.14
26	MPI-ESM1-2-HR	r2i1p1f1	0.70	-0.07
27	MPI-ESM1-2-LR	r1i1p1f1	0.59	-0.31
28	MPI-ESM1-2-LR	r2i1p1f1	0.75	-0.41
29	UKWESM1-0-LL	r1i1p1f2	0.69	-0.30
30	UKWESM1-0-LL	r2i1p1f2	0.70	0.37
31	UKWESM1-0-LL*	r3i1p1f2	0.67	0.75

1. Mazdiyasni, O. *et al.* Increasing probability of mortality during Indian heat waves. *Sci. Adv.* **3**, e1700066 (2017).

2. Mishra, V., Mukherjee, S., Kumar, R. & Stone, D. A. Heat wave exposure in India in current, 1.5 ℃, and 2.0 ℃ worlds. *Environ. Res. Lett.* **12**, 124012 (2017).

3. Russo, S., Sillmann, J. & Fischer, E. M. Top ten European heatwaves since 1950 and their occurrence in the coming decades. *Environ. Res. Lett.* **10**, 124003 (2015).

4. Barros, V. R., Field, C. B. *et al.* Climate change 2014: impacts, adaptation, and vulnerability. part b: regional aspects (2014).

5. Hagenlocher, M. *et al.* Drought vulnerability and risk assessments: state of the art, persistent gaps, and research agenda. *Environ. Res. Lett.* **14**, 083002 (2019).

6. Vittal, H., Karmakar, S., Ghosh, S. & Murtugudde, R. A comprehensive India-wide social vulnerability analysis: highlighting its influence on hydro-climatic risk. *Environ. Res. Lett.* **15**, 014005 (2020).

7. Srivastava, A., Rajeevan, M. & Kshirsagar, S. Development of a high resolution daily gridded temperature data set (1969–2005) for the indian region. *Atmospheric Science Letters* **10**, 249–254 (2009).

8. Wu, S.-Y., Yarnal, B. & Fisher, A. Vulnerability of coastal communities to sea-level rise: a case study of cape may county, new jersey, usa. *Climate research* **22**, 255–270 (2002).

9. Karmakar, S., Simonovic, S. P., Peck, A., Black, J. *et al.* An information system for risk-vulnerability assessment to flood. *Journal of Geographic Information System* **2**, 129 (2010).

10. Sherly, M. A., Karmakar, S., Parthasarathy, D., Chan, T. & Rau, C. Disaster vulnerability mapping for a densely populated coastal urban area: an application to mumbai, india. *Annals of the Association of American Geographers* **105**, 1198–1220 (2015).

11. Cooper, W., Seiford, L. & Zhu, J. Data envelopment analysis. handbook on data envelopment analysis. international series in operations research & management science, serie 71, 573 pages (2004).

12. Sharma, T., Vittal, H., Karmakar, S. & Ghosh, S. Increasing agricultural risk to hydro-climatic extremes in India. *Environ. Res. Lett.* **15**, 034010 (2020).

13. Molteni, F. Atmospheric simulations using a gcm with simplified physical parametrizations. i: Model climatology and variability in multi-decadal experiments. *Climate Dynamics* **20**, 175–191 (2003).

14. Kucharski, F. *et al.* On the need of intermediate complexity general circulation models: A “speedy” example. *Bulletin of the American Meteorological Society* **94**, 25–30 (2013).

15. Hari, V., Pathak, A. & Koppa, A. Dual response of arabian sea cyclones and strength of indian monsoon to southern atlantic ocean. *Climate Dynamics* **56**, 2149–2161 (2021).

16. Suarez-Gutierrez, L., Milinski, S. & Maher, N. Exploiting large ensembles for a better yet simpler climate model evaluation. *Clim. Dyn.* **57**, 2557–2580 (2021).

17. Tokarska, K. B. *et al.* Past warming trend constrains future warming in cmip6 models. *Science advances* **6**, eaaz9549 (2020).

18. Huang, X. *et al.* South Asian summer monsoon projections constrained by the interdecadal Pacific oscillation. *Sci. Adv.* (2020). URL `https://www.science.org/doi/10.1126/sciadv.aay6546`.

19. Zhao, X. & Allen, R. J. Strengthening of the Walker Circulation in recent decades and the role of natural sea surface temperature variability. *Environ. Res. Commun.* **1**, 021003 (2019).

20. Chiang, J. C. H. & Vimont, D. J. Analogous Pacific and Atlantic Meridional Modes of Tropical Atmosphere–Ocean Variability. *J. Clim.* **17**, 4143–4158 (2004).
